# Frequency, socio-economic characteristics, and risk factors of oral cavity parasites in diabetes mellitus patients from Lorestan Province, Iran; a case-control study

**DOI:** 10.3389/fcimb.2025.1522670

**Published:** 2025-04-01

**Authors:** Leila Masoori, Parastoo Baharvand, Amal Khudair Khalaf, Behnoush Selahbarzin, Fatemeh Sakifar, Hossein Mahmoudvand

**Affiliations:** ^1^ Department of Medical Parasitology and Mycology, Lorestan University of Medical Sciences, Khorramabad, Iran; ^2^ Department of Community Medicine, Lorestan University of Medical Sciences, Khorramabad, Iran; ^3^ Department of Microbiology, College of Medicine, University of Thi-qar, Dhi Qar, Iraq; ^4^ Department of Pediatric Dentistry, Lorestan University of Medical Sciences, Khorramabad, Iran; ^5^ Student Research Committee, Lorestan University of Medical Sciences, Khorramabad, Iran; ^6^ Hepatitis Research Center, Lorestan University of Medical Sciences, Khorramabad, Iran

**Keywords:** *Entamoeba gingivalis*, *Trichomonas tenax*, PCR, oral health, frequency

## Abstract

**Background:**

Numerous studies identify diabetes mellitus (DM) as one of the most significant risk factors for the development of periodontal diseases (gum diseases). Individuals with diabetes experience gingival destruction more rapidly and severely due to the accumulation of microbial plaque in the mouth. *Entamoeba gingivalis* and *Trichomonas tenax* are parasites commonly found in the human oral cavity. This study aims to determine, the frequency, socio-economic characteristics, and risk factors of *E. gingivalis* and *T.tenax* in DM patients from Lorestan Province, Iran as a case-control study.

**Methods:**

The current case-control study involved 500 DM patients who were referred to health centers in Lorestan province, Iran between December 2022 and June 2024. Furthermore, a control group comprising 500 healthy persons without DM (non-DM) who were referred to health centers during the same study period was incorporated into the research. The prevalence of parasites in the oral cavity was determined using microscopic analysis and conventional polymerase chain reaction (PCR) techniques. A questionnaire was administered to collect demographic information, including age, gender, place of residence, education level, occupation, monthly income, tooth brushing practices, and mouthwash usage.

**Results:**

Out of a total of 500 DM patients, 136 (27.2%) and 146 (29.2%) patients had the oral cavity parasites (*E*. *gingivalis* and *T. tenax*) by microscopic and PCR analysis, respectively. While, in non-DM, 61 (12.2%) and 65 (13.0%) tested positive for parasites using microscopic and PCR methods, respectively (P<0.001). Among several factors, income (P = 0.001, OR = 5.491, 95% CI: 4.089 to 9.723), place of residence (P = 0.006, OR = 1.982, 95% CI: 1.222), education (P = 0.002, OR = 3.577 (1.618, 5.907)), and use mouthwash demonstrated a significant protective effect on the oral cavity parasites.

**Conclusion:**

This research for the first time in Iran highlighted a considerable prevalence of oral cavity parasites in DM patients in Lorestan province, Western Iran. Dental professionals should maintain a heightened awareness of these risk factors to effectively identify and address oral health challenges within this population, thereby reducing the incidence of oral diseases and infections.

## Introduction

Diabetes Mellitus (DM) is a prevalent and intricate metabolic disorder that disrupts carbohydrate metabolism, affecting about 830 million individuals globally, with a significant proportion residing in low- and middle-income countries (Martínez-Castelao, 2023). The primary issue in this condition is either a reduction in insulin production or tissue resistance to insulin's effects. The ultimate consequence of this abnormal state is elevated blood sugar levels, known as hyperglycemia, which is the most significant complication associated with the disease (Lutz, 2023). DM is categorized into two types: Type 1, characterized by a defect in insulin secretion, and Type 2, marked by a defect in insulin function (Kumar et al., 2020). Diabetes can occur at any age and is a chronic condition. Individuals with diabetes are at a higher risk for complications related to the cardiovascular system, kidneys, nervous system, eyes, and particularly oral health, compared to individuals without the disease (ElSayed et al., 2023). Oral manifestations of DM include periodontal disease, which occurs more frequently in individuals with diabetes than in healthy individuals and progresses at a faster rate (Rohani, 2019). Additionally, the healing and repair of wounds are delayed due to alterations in collagen metabolism within the tissues. Consequently, the likelihood of oral and dental infections, erythema, and significant swelling of the gums is also elevated in these patients (Kumari and Gnanasundaram, 2021).

Numerous studies identify DM as one of the most significant risk factors for the development of periodontal diseases (gum diseases) (Leena et al., 2022). Inflammatory gum disease is characterized by chronic inflammation resulting from bacterial infection. Individuals with diabetes experience gingival destruction more rapidly and severely due to the accumulation of microbial plaque in the mouth (Graves et al., 2020). Gum abscesses are also more prevalent among diabetics, a trend attributed to the compromised immune response in these individuals (Graves et al., 2020). Furthermore, the incidence of various clinical forms of oral candidiasis is higher in this population. In patients with poorly controlled type 1 diabetes, conditions such as zygomycosis and benign migratory glossitis may also arise (Rodrigues et al., 2019). Additionally, diffuse and painless bilateral swelling of the parotid glands, known as diabetic sialadenosis, can occur in both types of diabetes (Martínez-García and Hernández-Lemus, 2021).

*Entamoeba gingivalis* and *Trichomonas tenax* are parasites commonly found in the human oral cavity (Eslahi et al., 2021; Stoyanov et al., 2024). These organisms inhabit the surfaces of teeth, gums, and periodontal pockets near the cervical regions of the teeth, and they are rarely found in the tonsil crypts (Eslahi et al., 2021; Stoyanov et al., 2024). The transmission of these parasites occurs through the exchange of oral secretions, the use of shared utensils, contaminated hands, dental instruments, and kissing (Arpağ and Kaya, 2020). According to the previous studies, these parasites may contribute to dental issues such as tooth decay, gingivitis, periodontitis, and even respiratory tract infections (Bao et al., 2020; Yaseen et al., 2021). Investigations indicated that both *T. tenax* and *E. gingivalis* possess highly active proteolytic and collagenolytic enzymes (El Sibaei et al., 2012; Azmi et al., 2024). Numerous researchers have documented instances of oral infections involving *E. gingivalis, T. tenax*, and various *Candida* species in the oral cavities of diabetic patients (Santos and Roldán, 2023). Furthermore, these parasites appear to serve as reservoirs for anaerobic pathogenic bacteria (Santos and Roldán, 2023). Various investigations have been conducted to examine the oral colonization of these parasites in both healthy individuals and those suffering from periodontal disease, gingivitis, undergoing hemodialysis, or diagnosed with cancer, yielding a range of results (Santos and Roldán, 2023). Considering the importance of oral and dental hygiene, particularly for patients with diabetes, this study aims to determine, the prevalence, socio-economic characteristics, and risk factor of oral cavity parasites in diabetes mellitus patients from Iran.

## 2 Materials and methods

### 2.1 Participants and sampling

The current case-control study involved 500 DM patients who were referred to two main diabetes care centers in Lorestan Province, Iran between December 2022 and June 2024. The present study included patients diagnosed with type II DM who had a confirmed history of the condition and who were both willing and capable of providing written informed consent. Furthermore, a control group comprising 500 healthy persons without DM (non-DM) who were referred to health centers during the same study period was incorporated into the research. In the case group, a non-probability consecutive sampling method was employed during the specified time frame. Conversely, the control group was utilized a non-probability quota sampling method that aligns with the case group regarding age, gender, and distribution of place of residence. The exclusion criteria for the study included individuals who declined to participate, those who had recently used systemic antibiotics within the previous three months, and individuals with immune system deficiencies (e.g., Acquired immunodeficiency syndrome, cancer chemotherapy, and lupus). Additionally, individuals with a history or current presence of clinically significant chronic diseases, excluding diabetes mellitus, as well as those with conditions such as substance abuse, alcohol dependence, or psychiatric disorders, were also excluded from participation.

### 2.2 Ethical approval

The protocol was authorized by Lorestan University of Medical Sciences' Committee on the Ethics of Animal Experiments (Permit Number: IR.LUMS.REC.1401.245). This approval is reliant on the proposal/documents received by this committee on 2023.01-18.

### 2.3 Questionnaire, socio-economic, and the possible risk factors

Information and consent documents were developed and disseminated to the participants in both studies groups. Following this, a questionnaire was administered to collect demographic information, including age, gender, place of residence, education level, occupation, monthly income, tooth brushing practices, and mouthwash usage.

### 2.4 Collecting samples

Samples were obtained from participants for microscopic analysis by collecting two samples with sterile swabs from saliva and dental plaque. Furthermore, an additional saliva sample was preserved in tubes containing sterile normal saline for molecular testing purposes (Bao et al., 2020).

### 2.5 Parasitological study

After the preparation of smears on glass slides, the slides were subjected to staining procedures utilizing trichrome and Giemsa stains, and were subsequently analyzed under a light microscope (Ghabanchi et al., 2010).

### 2.6 Conventional polymerase chain reaction

DNA purification from saliva and dental plaque samples was performed utilizing a commercial kit (Parstous, Iran) in accordance with the manufacturer's instructions. The extracted DNA samples were subjected to conventional PCR analysis for the identification of oral parasites. Two specific sets of PCR primers were utilized to amplify and detect *E. gingivalis* (small ribosomal RNA (SrRNA) gene, F5′- GCGCATTTCGAACAGGAATGTAGA -3′ and R 5′-CAAAGCCTTTTCAATAGTATCTTCATTCA-3′) and *T. tenax* (18S ribosomal RNA gene, F5′-ATGACCAGTTCCATCGATGCCATTC-3′) and R5′-CTCCAAAGATTCTGCCACTAACAAG -3′). The thermal cycling protocol for PCR included an initial denaturation step of 5 minutes at 95°C, followed by 37 cycles comprising denaturation at 95°C for 30 seconds, primer annealing at 62°C for 1 minute, and elongation at 74°C for 60s. A final elongation step was performed for 10 minutes at 73°C (Selahbarzin et al., 2024). To ensure the integrity of DNA extraction and the PCR results, appropriate positive and negative controls were implemented to mitigate contamination. Positive controls consisted of patient samples containing motile *E. gingivalis* and *T. tenax*, which yielded the expected amplicon sizes, while nuclease-free water was utilized as the negative control. The resulting amplicons were visualized using 1% agarose gel electrophoresis.

### 2 Statistical analysis

The statistical analysis of the results was conducted utilizing SPSS version 24.0 (SPSS Inc., Chicago, IL, USA). Chi-square tests were employed to assess the differences in participant distribution between the case and control groups, as well as to evaluate the associations between independent and dependent variables. Variables that demonstrated a significant relationship with oral cavity parasites were further analyzed as potential risk factors. Variables with a p-value of less than or equal to 0.20 were incorporated into the final multivariate logistic regression model. To ascertain the likelihood of exposure to the oral parasites in DM patients compared to healthy individuals, odds ratios with a 95% confidence interval were calculated. P-values of less than 0.05 were deemed indicative of statistical significance.

## 3 Results

### 3.1 Prevalence of oral cavity parasites

**Figure 1:**
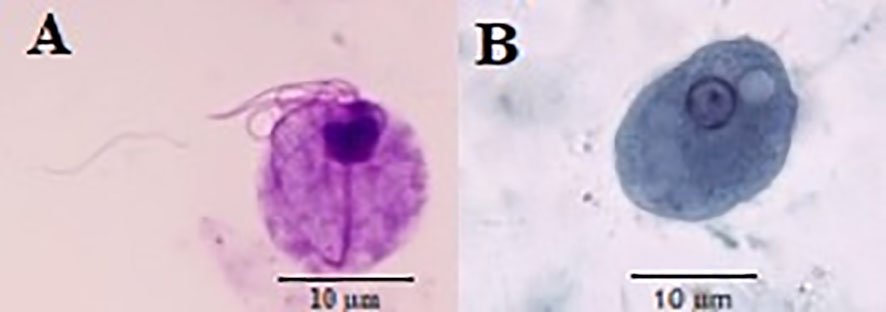
*Trichomonas tenax* **(A)** and *Entamoeba gingivalis* **(B)** stained by Giemsa and trichrome stains, respectively.

**Figure 2:**
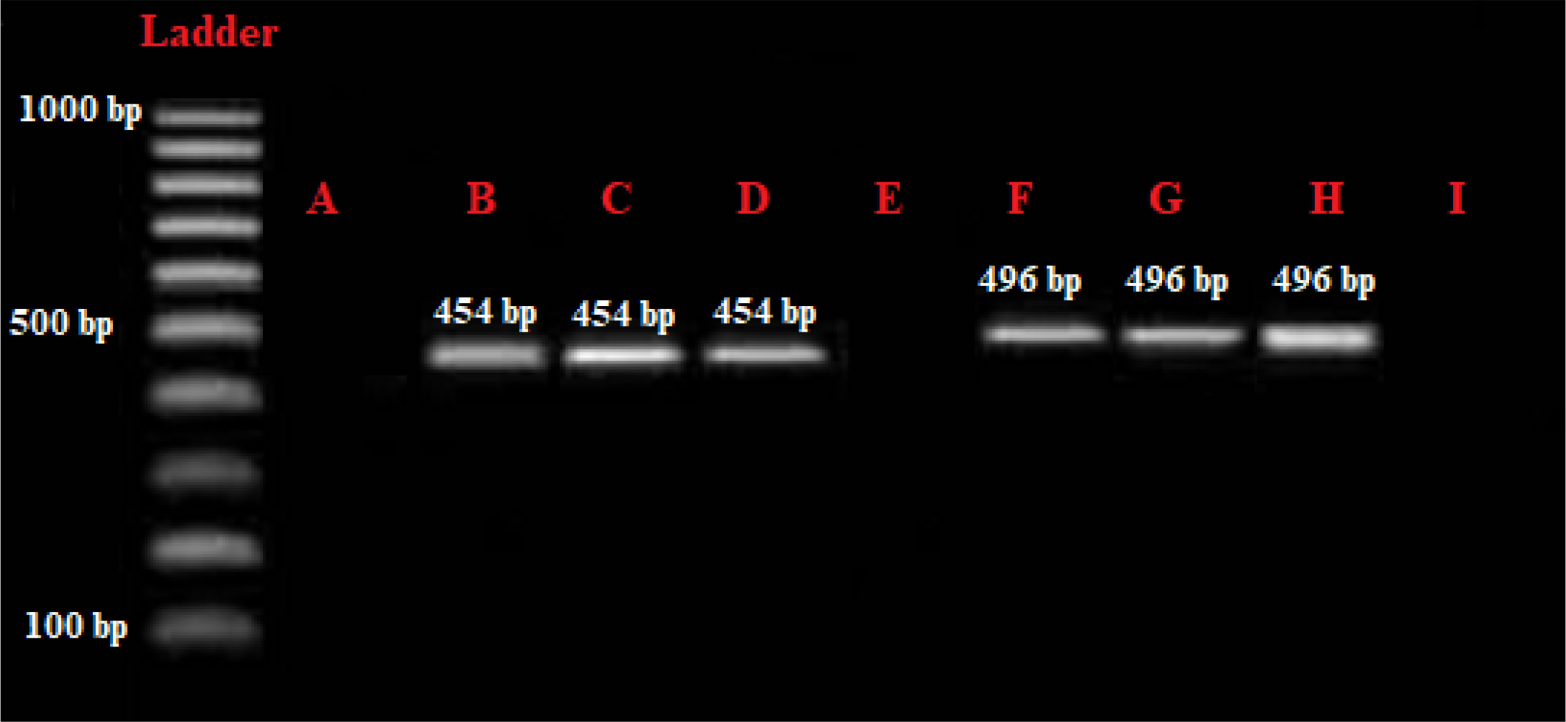
Gel electrophoresis assessment of the PCR products. **(A)** negative control; **(B)** Positive control of *Entamoeba gingivalis*, 454 bp; **(C, D)** positive samples of *E gingivalis*; **(E, I)** negative samples; **(F)** positive control of *Trichomonas tenax* positive control, 496 bp; **(G, H)** positive samples of *T. tenax*.

Out of a total of 500 DM patients, 136 (27.2%) and 146 (29.2%) patients had the oral cavity parasites (*E*. *gingivalis* and *T. tenax*) by microscopic (**Figure 1**) and PCR analysis (**Figure 2**). Within the positive samples, 101 (69.2%) and 44 (33.1%) DM patients were found to be infected with *E. gingivalis* and *T. tenax*, respectively; while one DM patient (0.7%) showed a mixed infection involving both *E. gingivalis* and *T. tenax.* In contrast, within the control group comprising 500 non-DM, 61 (12.2%) and 65 (13.0%) tested positive for parasites using microscopic and PCR methods, respectively. Among the positive samples from non-DM participants, 41 (63.1%) were identified as positive for *E. gingivalis*, while 24 (36.9%) tested positive for *T. tenax* (**Table 1**). These results suggest that the probability of exposure to oral cavity parasites in non-DM participants is significantly lower than that observed in the case group (*p<0.001*, OR = 0.411; CI= 0.274-0.539).

**Table 1:** A comparative analysis of the prevalence of oral cavity parasites among patients with diabetes mellitus (DM) and those without DM (non-DM) is conducted.

Group	Microscopic test		PCR method		OR	CI	P-value
	Positive No. (%)	Negative No. (%)	Positive No. (%)	Negative No. (%)			
DM	136 (27.2)	364 (72.8)	146 (29.2)	354 (70.8)	0.411	0.274-0.539	<0.001^*^
Non-DM	61 (12.2)	439 (87.8)	65 (13.0)	435 (87.0)	–	–	–

*p-value < 0.05, difference was statistically significant.

### 3.2 Demographic characterizations, socio-economic, and risk factors

In DM patients under the age of 30 yrs, 10 participants (20.0%) exhibited the presence of oral cavity parasites, while 40 participants (80.0%) did not show the presence of oral cavity parasites. Among the DM patients aged 30 to 60 years, 112 individuals (31.4%) tested positive for oral cavity parasites, in contrast to 245 individuals (68.6%) were negative positive for oral cavity parasites. In the DM patients aged over 60 years, 24 individuals (25.8%) were found to have oral cavity parasites, whereas 69 individuals (74.2%) did not have oral cavity parasites. The observed differences in the prevalence of oral cavity parasites across the various age groups were not statistically significant (*p=0.185*), indicating that there is no meaningful association between age and the presence of oral cavity parasites (p > 0.05). The results indicated that among 157 men DM patients, 42 men (26.8%) tested positive for oral cavity parasites, while 115 men (73.2%) tested negative. Among female DM patients, 104 individuals (30.3%) were positive for oral cavity parasites, whereas 239 individuals (69.7%) were negative for oral cavity parasites. The observed difference in the prevalence of oral cavity parasites between male and female participants was not statistically significant (*p=0.240*); demonstrating that there is no significant association between gender and the presence of oral cavity parasites (*p>0.05*). It was found that among DM patients who living in urban areas, 109 individuals (33.7%) tested positive for such infections, while 214 individuals (66.3%) tested negative. Conversely, DM patients who living in rural areas, 37 individuals (20.9%) were positive for oral cavity parasites n infections, and 140 individuals (79.1%) were negative. The observed difference in the prevalence of oral cavity parasites between urban and rural residents was statistically significant (*p=0.002*). This indicates a significant association between the residence and the prevalence of oral cavity parasites n *(p<0.05*). Among unemployed and employed DM patients, it was found that 113 unemployed patients (29.1%) tested positive for the oral cavity parasites. In contrast, among the employed DM patients, 33 out of 112 individuals (29.5 %) were positive for oral cavity parasites. The statistical analysis revealed that the difference in prevalence rates between the two occupational groups was not significant (*p=0.516*). This indicates that there is no statistically significant association between occupation and the presence of oral cavity parasites (*p>0.05*) (**Table 2**). In a similar vein, the analysis revealed no significant correlation between age (*p=0.185*), gender (*p=0.240*), and employment status (p=0.208) with the prevalence of oral cavity parasites among non-DM participants. Conversely, a significant association was found between the participants' place of residence and the prevalence of oral cavity parasites in the non-DM group (*p<0.002*) (**Table 3**). Among DM patients with illiteracy, 21 participants (39.6%) exhibited the presence of oral cavity parasites; while, in the DM patients with a diploma or lower educational attainment and DM patients with education exceeding a diploma, 104 individuals (30.2%) and 21 individuals (20.4%) tested positive for oral cavity parasites, respectively. The observed differences in the prevalence of oral cavity parasites across the various educational groups in DM patients were statistically significant (*p=0.033*), indicating a significant association between educational level and the presence of oral cavity parasites infection (*p<0.05*) (**Table 2**). However, no significant relationship was identified between the education level and the prevalence of both oral cavity parasites in the and non-DM participants (*p=0.564*) (**Table 3**). Concerning the prevalence of oral cavity parasites among DM patients with varying income levels, it was found that among those earning less than $250, 93 individuals (23.3%) tested positive for oral cavity parasites. In the DM patients with incomes ranging from $300 to $600, 46 individuals (68.7%) and among those with incomes exceeding $600, 7 individuals (21.2%) tested positive for oral cavity parasites, respectively. The observed differences in the prevalence of parasites in the oral cavity across the various income groups were statistically significant (*p=0.01*); indicated that there is a significant relationship between income and infection with oral cavity parasites (*p<0.05*). Similarly, a meaningful link was reported between income levels and the incidence of oral cavity parasites among non-DM participants (*p=0.001*).

**Table 2:** Frequency of oral cavity parasites in diabetes mellitus patients from Western Iran based on the demographic characterizations and the related risk factors.

Variables	Oral cavity parasites		*P-value
	Positive No. (%)	Negative No. (%)	
Age			
>30 yrs	10 (20.0)	40 (80.0)	*0.185*
30- 60 yrs	112 (31.4)	245 (68.8)	
60< yrs	24 (25.8)	69 (74.2)	
Gender			
Men	42 (26.8)	115 (73.2)	*0.240*
Women	104 (30.3)	239 (69.7)	
Residence			
Urban	119 (33.7)	214 (66.3)	0.002*
Rural	37 (20.9)	140 (79.1)	
Education			
illiterate	21 (39.6)	32 (60.4)	0.03*
≤Diploma	104 (30.2)	240 (69.8)	
>Diploma	21 (20.4)	82 (79.6)	
Occupation			
Unemployed	113 (29.1)	275 (70.1)	*0.516*
Employed	33 (29.5)	79 (70.5)	
Income			
<300 USD	93 (23.3)	307 (76.7)	0.001*
300-600 USD	46 (68.7)	21 (31.3)	
600< USD	7 (21.2)	26 (79.8)	
Tooth brushing			
No	112 (28.2)	285 (71.8)	*0.202*
Yes	34 (33.0)	69 (67.0)	
Mouthwash			
No	141 (31.3)	312 (68.7)	0.000*
Yes	5 (10.6)	42 (89.4)	

*p-value<0.05 significant difference by Chi-square analysis.

**Table 3:** Frequency of oral cavity parasites in healthy participants (non-diabetes mellitus) from Western Iran based on the demographic characterizations and the related risk factors.

Variables	Oral cavity parasites		*P-value
	Positive No. (%)	Negative No. (%)	
Age			
>30 yrs	5 (16.1)	25 (83.9)	*0.413*
30- 60 yrs	50 (12.7)	344 (87.3)	
60< yrs	10 (13.5)	64 (76.5)	
Gender			
Men	20 (15.8)	106 (75.2)	*0.231*
Women	45 (12.1)	329 (87.9)	
Residence			
Urban	56 (15.7)	301 (84.3)	0.000*
Rural	9 (6.3)	134 (93.7)	
Education			
illiterate	4 (12.4)	28 (87.8)	*0.564*
≤Diploma	52 (13.2)	342 (86.8)	
>Diploma	9 (12.1)	65 (87.9)	
Occupation			
Unemployed	45 (12.7)	309 (87.3)	*0.208*
Employed	20 (13.7)	126 (86.3)	
Income			
<300 USD	60 (14.3)	307 (76.7)	0.001*
300-600 USD	4 (6.3)	21 (31.3)	
600< USD	1 (5.5)	26 (79.8)	
Tooth brushing			
No	50 (13.8)	312 (86.2)	*0.246*
Yes	15 (10.8)	123 (89.2)	
Mouthwash			
No	64 (13.3)	417 (86.7)	0.002*
Yes	1 (5.3)	18 (94.7)	

*p-value <0.05 significant difference by Chi-square analysis.

The results showed that that 112 (28.2%) out of 397 DM patients who did not engage in regular tooth brushing tested positive for these oral cavity parasites. In non-DM participants who did not engage in regular tooth brushing, 50 individuals (13.8%) tested positive for the oral cavity parasites, while the rest were negative. The observed difference in the prevalence of oral cavity parasites and the tooth brushing among participants was not statistically significant in the DM (*p=0.202*) and non-DM participants (p=0.246). Oral cavity parasites were detected in 141 DM patients (31.1%) who did not use mouthwash, while, among non-DM patients who did not use mouthwash, 64 individuals (43.3%) were positive for oral cavity parasites. An examination of tooth brushing practices demonstrated a notable correlation between the frequency of mouthwash using and the occurrence of both *E. gingivalis* and *T. tenax* in individuals with DM (p<0.001) as well as in those without DM (*p=0.002*). This indicates a significant relationship between the use of mouthwash and parasites n infection in the oral cavity (*p<0.001*) (**Tables 2**, **3**).

### 3.3 Assessment of risk factors by regression analysis

Among several factors, income, place of residence, education, and use mouthwash demonstrated a significant predictive effect on the oral cavity parasites (*p<0.05*). Patients with an income of lower than 600 USD had a higher probability of oral cavity parasites compared to those with an income of > 600 USD (*p=0.001*, OR = 5.491, 95% CI: 4.089 to 9.723). The probability of oral cavity parasites was 1.98 times higher in patients who living in urban areas compared to rural regions (*p=0.006*, OR = 1.982, 95% CI: 1.222). Patients who were not literate had a 3.57 times greater chance of being infected with oral cavity parasites compared to patients with less than and equal to a diploma (*p= 0.002*, OR = 3.577 (1.618, 5.907)). Patients with a diploma education had 1.84 times the likelihood of being infected with oral cavity parasites compared to those who were higher diploma (*p=0.037*, OR = 1.864 [1.039 - 3.346]). Furthermore, DM patients who use mouthwash demonstrated a protective factor of oral cavity parasites [*p=0.001*, OR=0.016 (0.005-0.048)] (**Table 4**).

**Table 4:** Multiple logistic regression analysis for evaluation of the risk factors of oral cavity parasites in diabetes mellitus patients from Western Iran.

Variable	Univariable analysis		Multivariable analysis	
	Odds ratio (95% CI)	P-value	Odds ratio (95% CI)	P-value
Age	1.051 (0.730-1.515)	0.788	1.269 (0.593-2.716)	*0.539*
Gender	1.191 (0.781-1.817)	0.416	1.079 (0.672-1.734)	*0.753*
Residence	0.519 (0.338-0.797)	0.003*	4.546 (1.433-9.419)	0.010*
Education	1.610 (1.123-2.307)	0.010*	1.982 (1.222-3.215)	0.006*
Income	1.772 (1.291-2.431)	0.000*	5.491 (4.089-9.723)	0.000*
Occupation	1.017 (0.641-1.613)	0.944	1.164 (0.696-1.946)	*0.562*
Tooth brushing	1.254 (0.787-1.997)	0.341	1.158 (0.684-1.959)	*0.585*
Mouthwash	0.033 (0.013-0.082)	0.000*	0.016 (0.005-0.0480	0.000*

*p-value <0.05 significant difference.

## 4 Discussion

Oral manifestations of DM encompass periodontal disease, which is observed to occur with greater frequency in individuals with diabetes compared to those without the condition and exhibits a more rapid progression (Rohani, 2019). Previous researches indicated that *T. tenax* and *E. gingivalis* may play a role in the development of dental diseases, including gingivitis, periodontitis, and potentially respiratory tract infections (Eslahi et al., 2021). Given the significance of oral and dental hygiene, especially in patients with diabetes, this study seeks to investigate, for the first time, the prevalence of the oral cavity parasites and the related risk factors among DM patients from Western Iran.

Our study showed that 136 (27.2%) and 146 (29.2%) DM patients had the oral cavity parasites by microscopic and PCR analysis. Among the positive samples, 101 (69.2%) and 44 (33.1%) DM patients were found to be infected with *E. gingivalis* and *T. tenax*, respectively; while one DM patient (0.7%) showed a mixed infection involving both *E. gingivalis* and *T. tenax.* In contrast, within the control group comprising 500 non-DM, 61 (12.2%) and 65 (13.0%) tested positive for parasites using microscopic and PCR methods, respectively. Previous studies have examined the prevalence of oral cavity parasites among various populations in the current study area. These populations include individuals diagnosed with periodontitis (24 cases, 31.6%) in 2018, those suffering from dental caries (39 cases, 27.8%) in 2018, patients undergoing hemodialysis (20 cases, 27.4%) in 2023, pregnant women (46 cases, 23.0%) in 2022, children with intellectual disabilities (92 cases, 42.8%) in 2024, children with malignancies (28 cases, 31.1%) in 2023, and healthy children (117 cases, 17.7%) in 2024 (Derikvand et al., 2018; Mahmoudvand et al., 2018; Azadbakht et al., 2022; Azadbakht et al., 2023; Kooshki et al., 2023; Kooshki et al., 2024; Selahbarzin et al., 2024). In line with our results, Fadhil Ali Malaa et al. (2022) reported that the prevalence of *E. gingivalis* and *T. tenax* in DM patients from Iraq was 20.0% and 18.9%, respectively by microscopic analysis (Fadhil Ali Malaa et al., 2022). Another study conducted by Dybicz et al. (2018) showed that among 92 DM patients from Poland, 13 (14.1%) patients were positive for *T. tenax* by PCR examination (Dybicz et al., 2018). Nocito Mendoza et al. (2003) reported that the prevalence of *E. gingivalis* and *T. tenax* among 50 DM patients from Spain was 91.0% and 32.0%, respectively, by direct optical microscopy analysis (Nocito Mendoza et al., 2003). The variation in the prevalence of these oral cavity parasites is likely affected by factors including the characteristics of the study population, the size of the sample, the methodologies utilized in the research, health behaviors, and regional cultures.

Researches have shown that women tend to have more positive attitudes towards dental visits, demonstrate greater oral health knowledge, and practice better oral health habits than men (Lipsky et al., 2021). Nevertheless, our findings revealed no significant correlation between gender and the prevalence of oral cavity parasites in DM patients. Current evidence indicates that oral diseases disproportionately impact certain population subgroups characterized by limited economic resources, low educational attainment, inadequate access to dental care, and diminished social influence or political capital (Pronk et al., 2024). Our results showed that patients with an income of lower than 600 USD had 5.941 times higher probability of oral cavity parasites compared to those with an income of > 600 USD. While, we found that DM patients who were not literate had a 3.57 times greater chance of being infected with oral cavity parasites compared to patients with higher than and equal to a diploma. In a comparable manner, Selahbarzin et al. demonstrated a notable correlation between monthly family income (<200 USD) and the prevalence of oral cavity parasites in children with intellectual disabilities residing in Lorestan Province, Iran (Selahbarzin et al., 2024).

Since over fifty percent of the world's population resides in urban areas, the health disparities observed within these environments largely reflect the inequities present in economic, social, and living conditions that have characterized many societies as a result of urbanization (Kjellstrom and Mercado, 2008). Accordingly, we found that the likelihood of oral cavity parasites was found to be 1.98 times greater in DM patients residing in urban areas in comparison to those living in rural regions. In line with our results, Kooshki et al. (2023) have indicated a significant association between urban residency and the prevalence of oral cavity parasites among pediatric cancer patients in Lorestan province, Iran (Kooshki et al., 2023).

Standard guidelines for the maintenance of daily oral hygiene encompass tooth brushing and interdental cleaning (McGrath et al., 2023). Research literature suggests that the incorporation of a mouthwash as an adjunctive measure offers advantages that extend beyond mechanical cleaning methods. Antimicrobial mouthwashes are recognized for their ability to diminish dental plaque biofilm, thereby contributing to the prevention of oral diseases related with plaque accumulation (McGrath et al., 2023). Our findings revealed that DM patients who use mouthwash demonstrated a reduced risk of oral cavity parasites. Consistent with our findings, Kooshki et al. (2023) observed a significant association between the use of mouthwash and the prevalence of oral cavity parasites in pediatric patients with malignancies in Western Iran (Kooshki et al., 2023). The strengths of this study encompass its status as the inaugural investigation into the prevalence of these oral parasites among diabetic individuals in Iran. Additionally, it incorporates both microscopic and molecular analyses to detect the presence of these parasites, as well as an assessment of related risk and economic factors. The primary limitations of this study include the absence of sample collection from patients with type I diabetes and the need to explore additional socioeconomic and risk factors. These limitations will be addressed in our future research endeavors.

## 5 Conclusion

This research highlighted a considerable prevalence of oral cavity parasites in DM patients in Lorestan province, Western Iran. It is essential to identify the primary risk factors and related socio-economic determinants linked to these parasites, particularly insufficient dental hygiene practices, to improve public and oral health initiatives for DM patients. Therefore, dental professionals should maintain a heightened awareness of these risk factors to effectively identify and address oral health challenges within this population, thereby reducing the incidence of oral diseases and infections.

## Data Availability

The original contributions presented in the study are included in the article/supplementary material. Further inquiries can be directed to the corresponding author.
